# A Linear Affair: Blaschkoid Pattern in Lichen Planus Pigmentosus

**DOI:** 10.7759/cureus.83388

**Published:** 2025-05-03

**Authors:** Jebisha J B, Arun Vignesh, Murugan Sundaram, Sudha Rangarajan, Adikrishnan Swaminathan

**Affiliations:** 1 Dermatology, Venereology and Leprosy, Sri Ramachandra Institute of Higher Education and Research, Chennai, IND

**Keywords:** blaschko lines, dermoscopy findings, lichen planus pigmentosus, linear variant, pigmentary disorders

## Abstract

Lichen planus pigmentosus (LPP) is an acquired disorder of hyperpigmentation. Atypical presentations, such as linear or blaschkoid distribution, are exceedingly rare and may pose diagnostic challenges. We report a case of a 32-year-old male with pruritic, hyperpigmented, slate-gray to brown macules and patches distributed linearly along Blaschko’s lines on the right upper back and upper limb. The diagnosis of LPP was corroborated by clinical appearance, dermoscopic examination, and histopathological analysis. Recognition of such unusual patterns is essential for accurate diagnosis and to differentiate from other linear pigmentary disorders.

## Introduction

Lichen planus pigmentosus (LPP) is a variant of lichen planus [1]. It is clinically characterized by the hyperpigmented macules varying from slate-gray to dark brown, with or without pruritus, mainly over the photo-exposed areas. It is more common in individuals with darker skin types and typically manifests after the age of 30, with a higher incidence in females [2]. LPP is typically asymptomatic, with pruritus being less pronounced than in classical lichen planus. This disorder is characterized by a gradual onset and a chronic, prolonged course [3]. Linear LPP is a variant that can follow Blaschko’s lines. These lines reflect the pattern of epidermal cell migration during embryonic development [1]. This variant is not only uncommon but also underrecognized in clinical practice, with limited cases reported in the literature. The linear configuration along Blaschko’s lines introduces a novel dimension to our understanding of LPP pathogenesis, raising important considerations about embryonic cutaneous mosaicism and its role in disease localization.

## Case presentation

A 32-year-old male patient presented to our outpatient department with an eight-month history of multiple dark lesions over his right upper back extending to his right upper limb. The lesions were occasionally pruritic. The pigmentation initially started over the right upper back and gradually progressed to involve the right upper arm in a linear pattern. History did not reveal any significant trigger.

**Figure 1:**
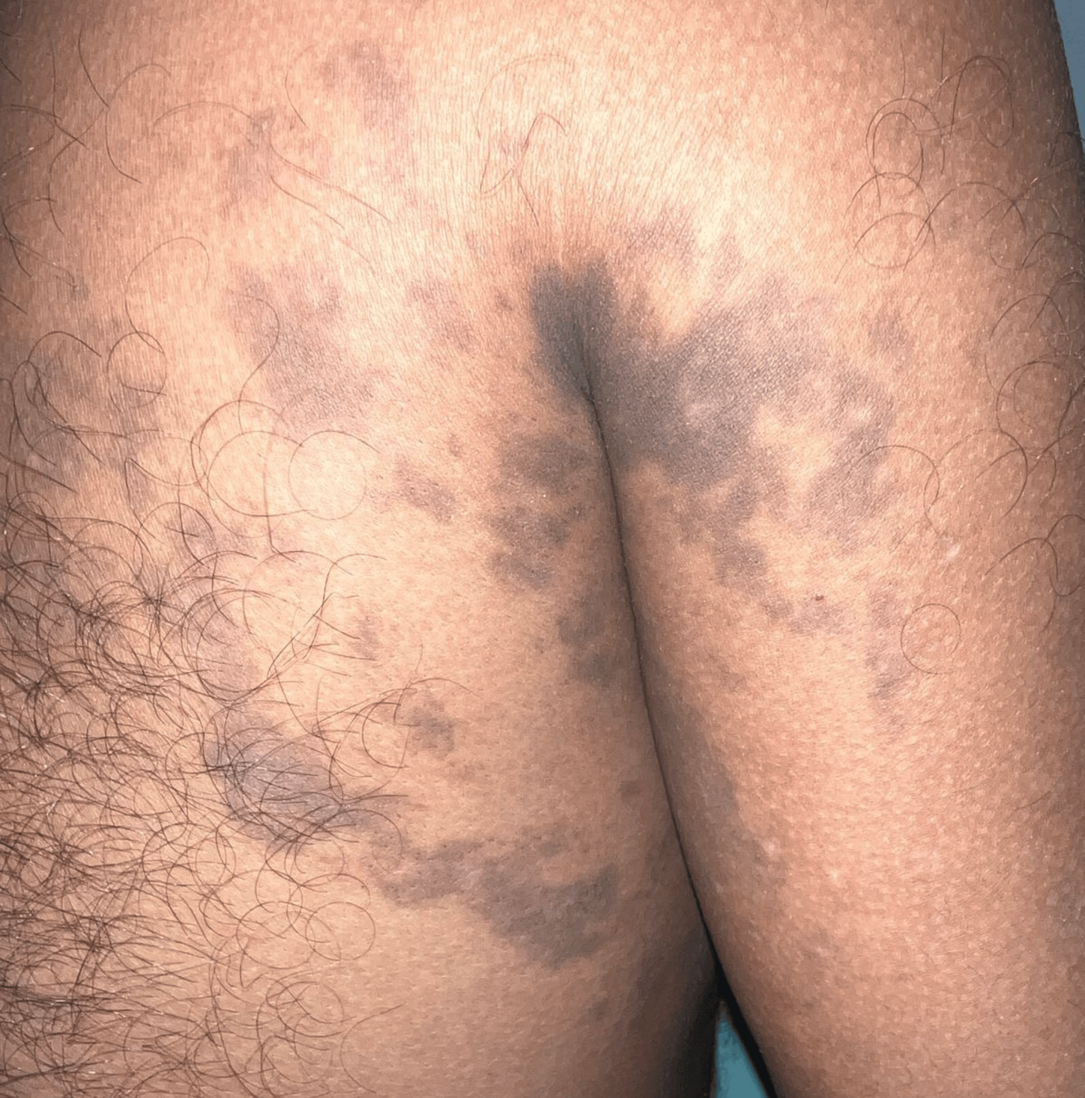
Hyperpigmented slate-gray to brown macules arranged in a curvilinear pattern along Blaschko’s lines over the right upper back and shoulder

**Figure 2:**
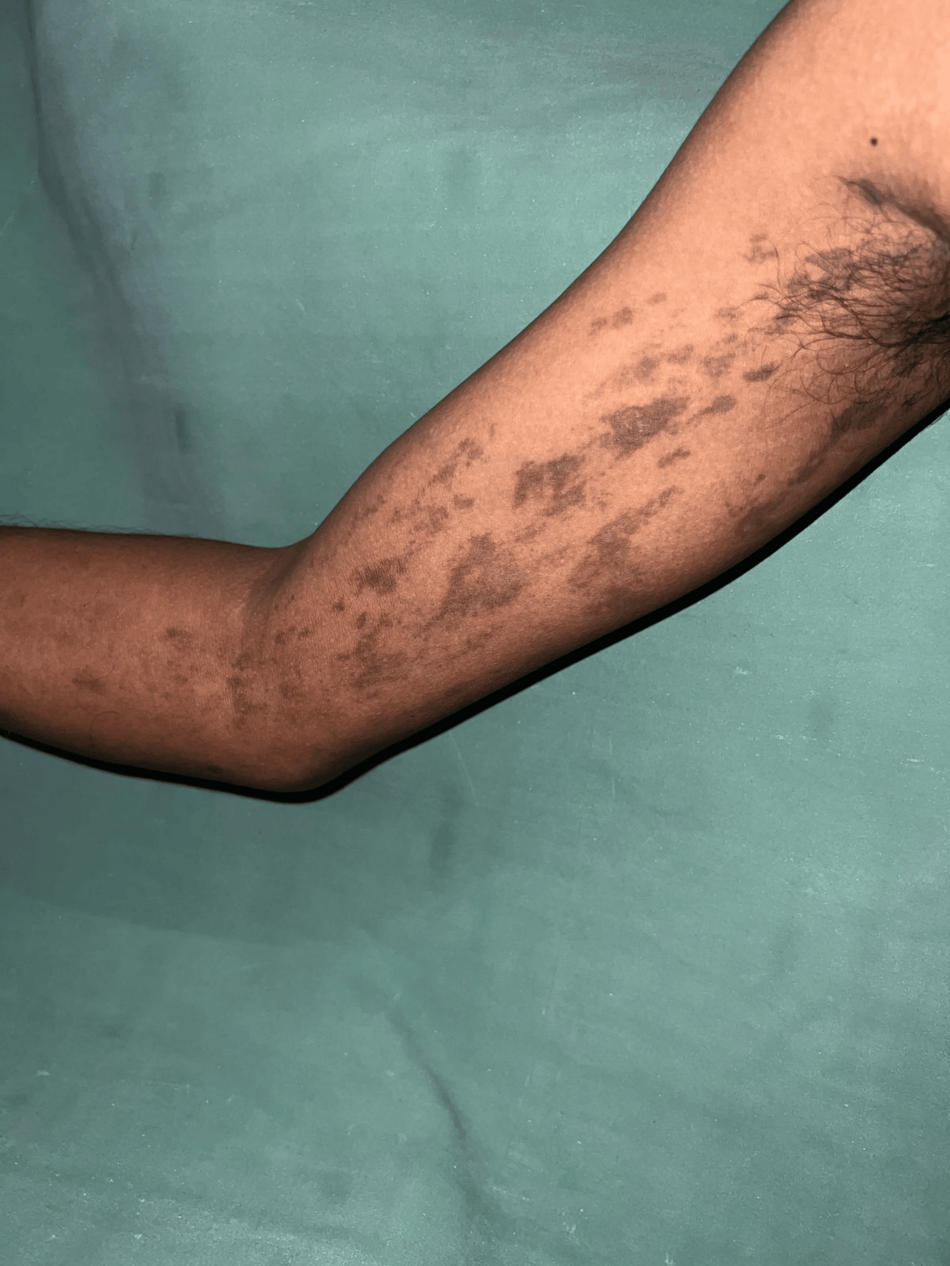
Lesions extending linearly across the flexor aspect of the right upper limb

On cutaneous examination, multiple hyperpigmented, slate gray to brown macules and patches, ranging in size from approximately 0.5 × 0.5 cm to 8.0 × 3.0 cm, were noted in a linear pattern following Blaschko’s lines over the right upper back, right axilla, and over both the extensor and flexor aspects of the right arm, elbow, and forearm (Figure 1, Figure 2). There were no similar lesions elsewhere. Mucous membrane and nail examinations were normal.

**Figure 3:**
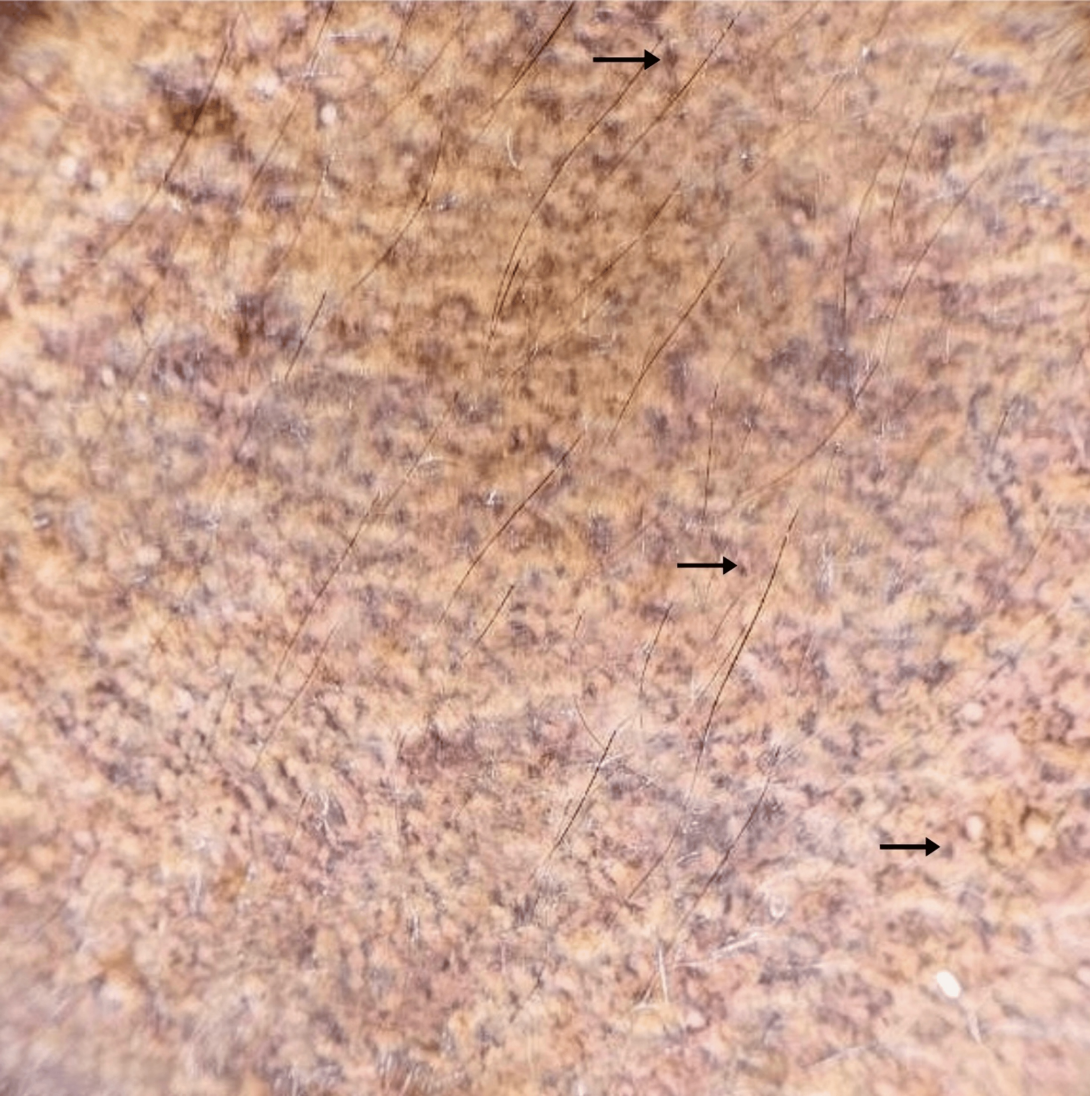
Dermoscopic examination (10x magnification) Brown to bluish gray dots and globules arranged in a reticular pattern (black arrows).

**Figure 4:**
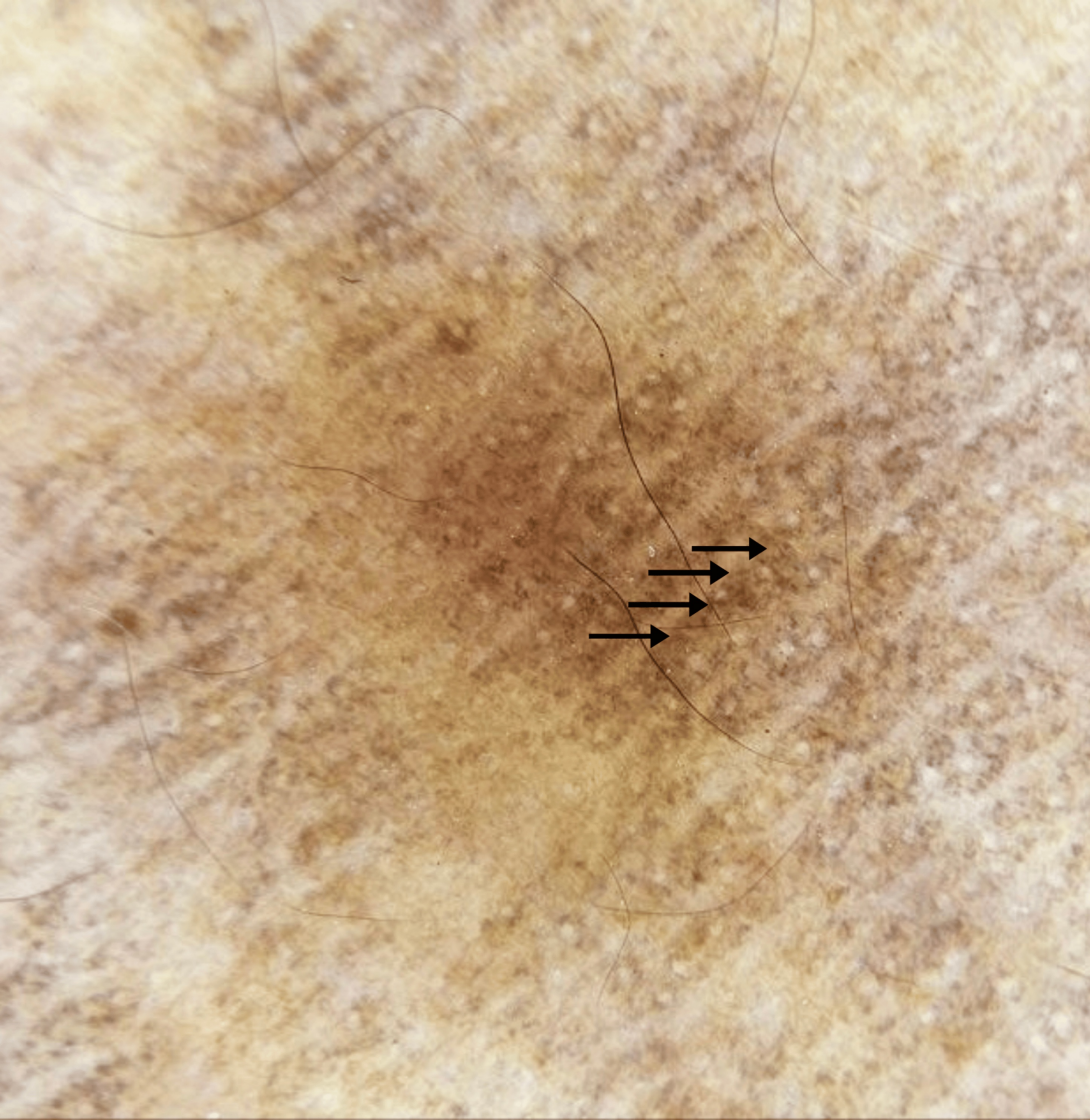
Dermoscopic examination (10x magnification) Brown dots and globules arranged in a hem-like pattern (black arrows).

**Figure 5:**
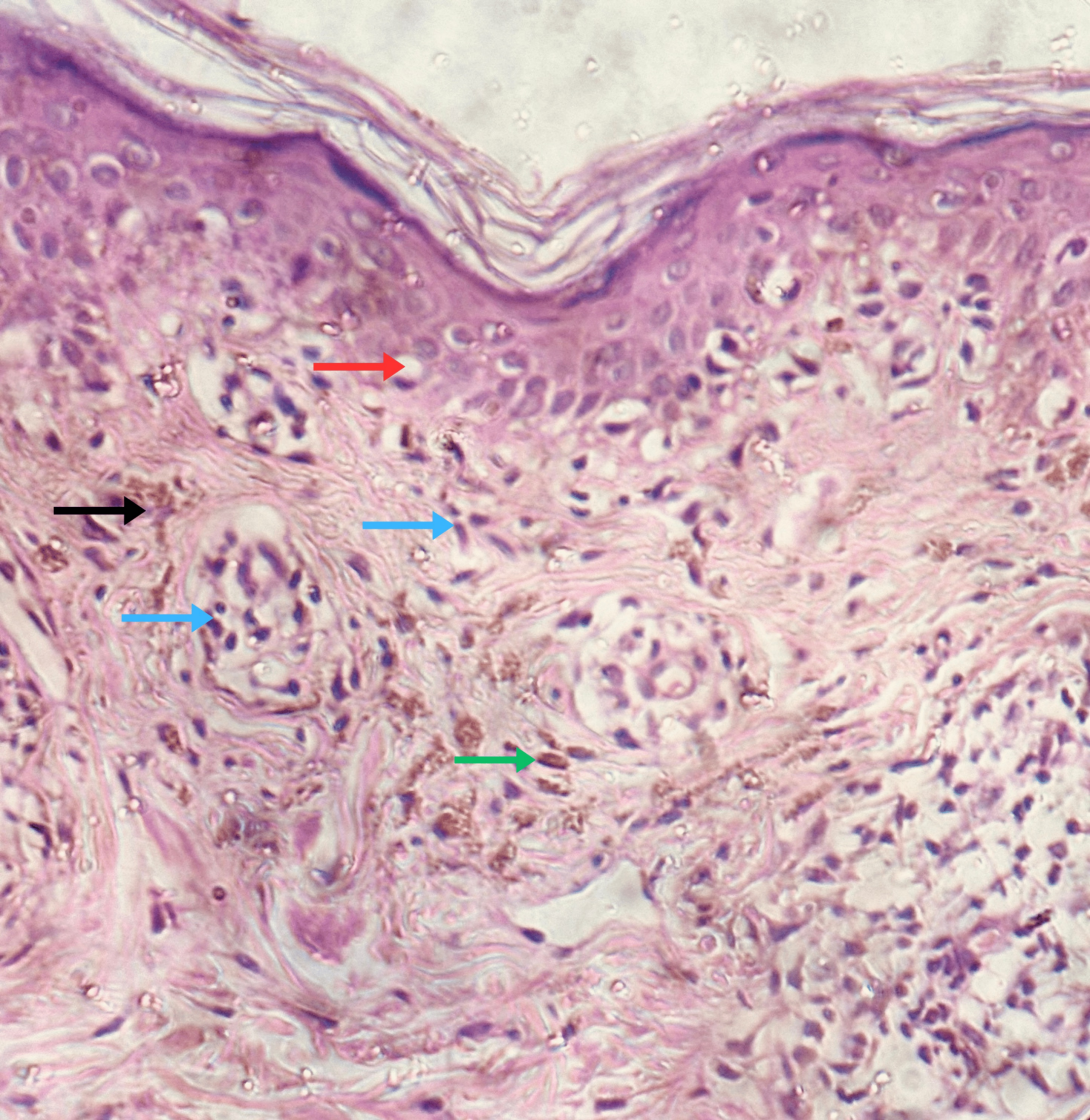
Histopathological examination (H&E stain, 400x) Atrophic epidermis, basal vacuolar degeneration (red arrow), superficial and perivascular lymphocytic infiltrates (blue arrows), melanin incontinence (black arrow), and pigment-laden macrophages (green arrow) in the upper dermis.

Dermoscopy showed perifollicular brown and blue-gray dots and globules in a hem-like pattern and in a reticular pattern (Figure 3, Figure 4). Histopathological examination showed atrophic epidermis, basal vacuolar changes, upper dermal lymphocytic infiltrates with perivascular involvement, melanin incontinence, and pigment-laden macrophages in the papillary dermis (Figure 5). A diagnosis of Blaschkoid LPP was made. The patient was initiated on a treatment regimen consisting of broad-spectrum sunscreen, topical mometasone furoate 0.1% cream, topical tacrolimus 0.1% ointment, and oral acitretin at a dose of 0.5 mg/kg/day.

## Discussion

LPP is recognized as a distinct clinical entity from classical lichen planus. It typically manifests as chronic, asymptomatic, or mildly pruritic, hyperpigmented, discrete, oval macules. These lesions usually coalesce into larger patches of hyperpigmentation, predominantly involving sun-exposed areas such as the face, trunk, and upper limbs. Although the oral mucosa can occasionally be affected, the palms, soles, and nails are typically unaffected [2].

LPP is more commonly seen in individuals with darker skin types. It affects both sexes, a higher prevalence has been noted among females [2]. LPP is considered a type IV hypersensitivity reaction to an unidentified antigen [4]. Sunlight is regarded as a primary etiological factor in the development of LPP [2]. Additional contributing factors may include photosensitizing agents such as mustard oil and amla oil, mechanical friction, aftershave lotions, fragrances, and cosmetic products, including hair dye and kumkum [3,4]. Furthermore, a potential association with the hepatitis C virus has been reported in the literature [5].

Various forms of LPP, such as diffuse, reticular, blotchy, follicular, inversus, and linear variants, have been documented [3,5]. Blaschkoid LPP represents an uncommon clinical variant. Lines of Blaschko are hypothesized to correspond to the migration of ectodermal and neuroectodermal cells during embryogenesis [1]. The manifestation of LPP along the lines of Blaschko indicates a possible embryonic origin, suggesting that somatic mosaicism was established during early development. This may result in site-specific antigenic differences, triggering a localized T-cell-mediated immune response [6].

Dermoscopy of LPP typically reveals slate-gray or blue-gray dots and globules, suggesting dermal melanophages. Other findings include a lack of Wickham striae, absence of vascular patterns, and pigment around follicles and eccrine openings. Dermoscopy showed varying patterns such as hem-like, arcuate, incomplete reticular, and complete reticular, indicating different stages of pigment incontinence, with more advanced cases showing reticular configurations [7].

Histopathological features include atrophic epidermis with basal cell vacuolar degeneration and variable band-like or perivascular lymphocytic infiltration in the upper dermis along with melanin incontinence. In early stages, lesions typically exhibit a band-like lymphocytic infiltrate, whereas in late stages, the lymphocytic infiltrate is predominantly perivascular [4]. Immune deposits are observed in approximately 15% of cases. Findings include IgM (occasionally IgG), C3, and fibrinogen in colloid bodies, with linear IgM and C3 along the basement membrane zone [2,5].

Other clinically similar entities to be considered include erythema dyschromicum perstans (EDP), linear fixed drug eruption, incontinentia pigmenti, and linear and whorled nevoid hypermelanosis [8]. Dermoscopic features help differentiate these entities from LPP. Small gray-blue dots and globules on a bluish background, reflecting deep dermal pigment and melanophages without follicular involvement, are seen in EDP [7]. Linear fixed drug eruption reveals brown to gray pigment dots and globules, reflecting melanin depth from pigment incontinence [9]. Incontinentia pigmenti in its pigmentary stage displays gray dots, with no appendageal structures [10]. Linear and whorled nevoid hypermelanosis presents as linear or whorled brown streaks in a parallel pattern following Blaschko’s lines [11].

The natural course of LPP remains uncertain, with some cases resolving spontaneously and others persisting for years [4]. Management includes minimizing sun exposure by using broad-rimmed hats, umbrellas, and broad-spectrum sunscreens [2].

Among topical therapies, corticosteroids have demonstrated inconsistent efficacy, possibly due to variable depth and chronicity of pigmentation [4]. In contrast, topical calcineurin inhibitors, especially tacrolimus, have shown promising results by modulating T-cell-mediated inflammation without the adverse effects of long-term corticosteroid use [5]. Depigmenting agents such as azelaic acid, kojic acid, and hydroquinone have been utilized with variable outcomes [12]. Additionally, topical ruxolitinib has emerged as a potentially effective and low-risk treatment option [13]. Phototherapy, particularly narrowband UVB, has shown potential in resolving lesions by reducing pro-inflammatory cytokines, inhibiting Langerhans cell-mediated antigen presentation, and suppressing T-cell-driven immune responses in the skin [14].

Systemic treatments such as corticosteroids, retinoids, and dapsone have shown significant improvement in patients with LPP [4]. Oral prednisolone has shown moderate efficacy in patients with LPP, while dapsone may help in arresting the progression of pigmentation [12,4]. A prospective, open-label pilot study of low-dose isotretinoin at 20 mg/day in LPP showed clinical improvement in 85.2% of patients, likely due to its anti-inflammatory and immunomodulatory effects, without significant side effects [15]. In a prospective study, oral tranexamic acid at 250 mg/day led to partial improvement in 10 of 20 LPP patients and resolved pruritus in all, with no side effects [16]. Furthermore, systemic agents such as tofacitinib and azathioprine have demonstrated remarkable clinical responses and efficacy in halting disease progression [17,18].

Procedural treatments such as superficial glycolic acid and Jessner’s peels have been effective in reducing pigmentation by inducing epidermal injury to promote keratinocyte turnover and melanophage clearance [19]. Combination therapy with topical tacrolimus and low-fluence 1064-nm Q-switched Nd:YAG laser has also achieved complete lesion clearance in certain cases [20].

## Conclusions

Owing to its rare blaschkoid pattern, this case was considered noteworthy and highlights the diversity of LPP presentations. Recognition of atypical variants is essential for accurate diagnosis and appropriate management. Given the potential for clinical overlap with other pigmentary disorders, an integrated approach utilizing clinical examination, dermoscopy, and histopathological evaluation is crucial in establishing a definitive diagnosis. Furthermore, awareness of the diverse morphological patterns of LPP can aid clinicians in differentiating it from mimickers and implementing targeted therapeutic strategies. Continued reporting of such uncommon presentations will enhance our understanding of the disease spectrum and contribute to refining management protocols for LPP.
